# Nitric Oxide Mediates the Stress Response Induced by Diatom Aldehydes in the Sea Urchin *Paracentrotus lividus*

**DOI:** 10.1371/journal.pone.0025980

**Published:** 2011-10-11

**Authors:** Giovanna Romano, Maria Costantini, Isabella Buttino, Adrianna Ianora, Anna Palumbo

**Affiliations:** 1 Laboratory of Functional and Evolutionary Ecology, Stazione Zoologica Anton Dohrn, Villa Comunale, Naples, Italy; 2 Laboratory of Cellular and Developmental Biology, Stazione Zoologica Anton Dohrn, Villa Comunale, Naples, Italy; Institute of Marine Research, Norway

## Abstract

Diatoms are ubiquitous and abundant primary producers that have been traditionally considered as a beneficial food source for grazers and for the transfer of carbon through marine food webs. However, many diatom species produce polyunsaturated aldehydes that disrupt development in the offspring of grazers that feed on these unicellular algae. Here we provide evidence that production of the physiological messenger nitric oxide increases after treatment with the polyunsaturated aldehyde decadienal in embryos of the sea urchin *Paracentrotus lividus*. At high decadienal concentrations, nitric oxide mediates initial apoptotic events leading to loss of mitochondrial functionality through the generation of peroxynitrite. At low decadienal concentrations, nitric oxide contributes to the activation of *hsp70* gene expression thereby protecting embryos against the toxic effects of this aldehyde. When nitric oxide levels were lowered by inhibiting nitric oxide synthase activity, the expression of *hsp70* in swimming blastula decreased and the proportion of abnormal plutei increased. However, in later pluteus stages nitric oxide was no longer able to exert this protective function: *hsp70* and *nitric oxide synthase* expression decreased with a consequent increase in the expression of *caspase-8*. Our findings that nitric oxide production increases rapidly in response to a toxic exogenous stimulus opens new perspectives on the possible role of this gas as an important messenger to environmental stress in sea urchins and for understanding the cellular mechanisms underlying toxicity during diatom blooms.

## Introduction

Diatoms are one of the largest and ecologically most significant groups of organisms on Earth, accounting for as much as 20% of global photosynthetic fixation of carbon (∼20 Pg carbon fixed per year) [1], which is more than all the world's tropical rainforests. These microscopic, unicellular algae have traditionally been considered as beneficial for the reproduction and development of plankton primary consumers, consisting mainly of small crustacean copepods that dominate the zooplankton, and in the transfer of carbon to higher trophic levels. The discovery that some diatom species produce polyunsaturated aldehydes (PUAs) with antiproliferative activity [2] has challenged this view [3] introducing a new perspective into plant-animal interactions and energy flux in marine food chains.

PUAs are the end-products of a lipoxygenase/hydroperoxide lyase metabolic pathway [4]–[7] initiated by damage to algal cells, as occurs through grazing by predators. Cell damage activates lipase enzymes, which liberate polyunsaturated fatty acids from cell membranes that are immediately oxidized and cleaved within seconds to form PUAs and a plethora of other metabolites collectively termed oxylipins. The specific type and quantity of oxylipins produced differs between diatom species and strains due to a variety of precursor polyunsaturated fatty acids and enzymes with variable effects on grazers. Similar wound-activated compounds are also found in terrestrial plants where they play a pivotal role in defense because of their antibacterial, wound healing and antiproliferative activity [8]. What remains unclear and still hotly debated in biological oceanography is the function of these molecules in the marine environment [3], [9].

PUAs can compromise embryonic and larval development in benthic organisms as well, by inhibiting fertilization processes, reducing larval fitness and inducing teratogenesis in several broadcast spawning species [10], [11]. For example, sea urchin gametes incubated in the diatom PUA decadienal (DD) showed impaired fertilization success due to inhibition of both sperm motility [12], [13] and pronuclear fusion [14]. Arrest of cell cleavage has been reported by various authors in both *Paracentrotus lividus* and *Sphaerechinus granularis* eggs treated with DD [2], [15], [16]. DD has been found to induce apoptotic events via caspase-3-like protease activity [17] and inhibit tubulin polymerization, DNA synthesis and cyclin B/Cdk1 kinase activity [14], leading to arrest in cell cycle progression in early embryos.

To better understand the mechanism by which PUAs affect sea urchin development we investigated the possible involvement of nitric oxide (NO), a well-known physiological messenger formed by the oxidation of L-arginine catalyzed by the enzyme nitric oxide synthase (NOS), which is endogenously produced during sea urchin development. NO is implicated in important processes occurring at fertilization in sea urchin, such as duration of the calcium transient, increase in NAD(P)H and H_2_O_2_ production and fertilization envelope hardening [18], [19]. However, there are no data on the possible involvement of NO in early development processes including cell cycle regulation and transcriptional gene regulatory networks that have been extensively studied and well characterized in this model organism [20]–[23]. On the contrary, in later developmental stages, NO has been shown to act as a signal during settlement and metamorphosis [24], [25]. To our knowledge no information is available on the role of NO in response to environmental stress in sea urchins. In other marine organisms, NO is considered an ancient cellular signal of environmental stress with elevated temperatures activating NO production in sponges [26]. Other stimuli such as salinity and light have also been shown to give rise to NO bursts in culture media of marine microalgae [27]. Recently, NO has been shown to be involved in coral bleaching by affecting the symbiotic relationship between corals and algae [28]–[30].

Here we show that this gas mediates the toxic effect of diatom PUAs on sea urchin *P. lividus* development: at high DD concentrations NO induces initial apoptotic events whereas at low DD concentrations, NO protects developing embryos against teratogenesis through the expression of specific genes such as *hsp70*.

## Materials and Methods

### Ethics Statement

*Paracentrotus lividus* (Lamarck) sea urchins were collected from a location that is not privately-owned nor protected in any way, according to the authorization of Marina Mercantile (DPR 1639/68, 09/19/1980 confirmed on 01/10/2000). The field studies did not involve endangered or protected species. All animal procedures were in compliance with the guidelines of the European Union (directive 609/86).

### Gamete collection

Sea urchins were collected during the breeding season by our fishermen in the Gulf of Naples, transported in an insulated box to the laboratory within 1 h after collection, and maintained in tanks with circulating sea water until testing. To induce gamete ejection, sea urchins were injected with 0.2 ml of 0.2 M acetylcholine (Sigma-Aldrich) through the peribuccal membrane. Eggs were washed with filtered sea water (FSW) and kept in FSW until use. Concentrated sperm was collected dry and kept undiluted at +4°C until use. Sperm to egg ratios were 100∶1 for both controls and treated embryos.

### Nitric oxide (NO) detection

NO detection was performed using 4-amino-5-methylamino-2′,7′-difluorofluorescein diacetate (DAF-FM-DA) (Molecular Probes), a fluorescent probe that is often used for imaging intracellular NO production in biological systems including sea urchins [18], [19], [31]. About 50 sea urchin eggs were incubated in the dark with 50 µM DAF-FM-DA in FSW (200 µl) for 20 min. Eggs were washed three times in FSW for an overall duration of 30 min to allow de-esterification of intracellular diacetate. Washed eggs were incubated for 10 min with 2-*trans*-4-*trans*-decadienal (DD) (Sigma–Aldrich) at concentrations ranging from 1 to 5 µg/ml in the absence or presence of 800 µM [2-(4-carboxyphenyl)-4,4,5,5-tetramethylimidazoline-1-oxyl-3oxide] (c-PTIO) (Alexis). DD stock solutions were prepared as recently described [11]. Control eggs were incubated for the same length of time in FSW. Eggs were fertilized as reported above. Acquisition of fluorescence started immediately and lasted for at least 20 min using a Zeiss- LSM 510 META confocal microscope in time lapse mode (Laser 488, emission filter: BP 500–550). To calculate changes in fluorescence for each sample we used the ImageJ program and calculated the difference between initial fluorescence prior to DD addition and after 20 min DD treatment. A selected region of interest was used to include the whole egg, with or without the fertilization envelope, and mean values were obtained from a number of eggs ranging from 4 to 6. Only fertilized eggs were considered for fluorescence measurements. One-way ANOVA with Tukey's post test was performed using GraphPad Prism version 4.00 for Windows (GraphPad Software, San Diego California USA).

### Mitochondrial functionality assay and apoptosis detection

To assess mitochondrial functionality, the fluorescent dye Mito Tracker (Molecular Probes) was used. This dye stains mitochondria in live cells and is accumulated inside the organelle depending on membrane potential. Eggs were incubated for 10 min in 5 µg/ml DD in the absence or presence of 10 µM [manganese (III) tetrakis (4-benzoic acid) porphyrin chloride] (MnTBAP) (Alexis). Eggs were then fertilized as reported above and after 20 min incubated in 200 µl of 2.5 µM Mito Tracker in FSW (stock solution: 1 mM in DMSO) in the dark. Control samples were run in parallel in FSW. Fluorescence was visualized at 50 min post-fertilization using a Zeiss-LSM 510 META confocal microscope (Laser 543, emission filter: BP 565–615).

To assess apoptosis, eggs were incubated for 10 min in 4 ml FSW containing DD, in the absence or presence of 800 µM of the NO scavenger c-PTIO added 10 min before DD. Eggs were fertilized as described above and the appearance of blebbing was evaluated at different time intervals by counting at least 200 embryos for each well using a light microscope (Zeiss Axiovert 135TV). Controls were performed in FSW. Experiments were conducted in triplicate using three egg groups collected from three different females.

### Teratogenic assay

Eggs were fertilized as described above and allowed to develop at 20°C in a controlled temperature chamber at 12∶12 light/dark cycle. Before fertilization, eggs were incubated for 10 min in DD or FSW (control) at the concentrations indicated in the text. Incubations with 1-(2-trifluoromethylphenil)imidazole (TRIM) (Alexis) or *N* ^G^-nitro-D-arginine (D-NA) (Alexis) or *N^ω^*-nitro-L-arginine (L-NA) (Sigma) were performed for 10 min before DD addition. Experiments were conducted in triplicate using three egg groups collected from three different females. After 48 h incubation, the percentage of dead and abnormal plutei were determined by counting at least 200 embryos for each well [32] using a light microscope (Zeiss Axiovert 135TV). Pictures were taken using a Zeiss Axiocam connected directly to the microscope. One-way ANOVA with Tukey's post test was performed using GraphPad Prism version 4.00 for Windows (GraphPad Software, San Diego California USA).

### RNA extraction and cDNA synthesis

About 30000 eggs in 200 ml FSW were treated for 10 min with 0.25 µg/ml DD and then fertilized. Incubation with 100 µM TRIM was performed for 10 min before DD addition. (Z)-1-[N-(3-Aminopropyl)-N-[4-(3-aminopropylammonio)butyl]-amino]diazen-1-ium-1,2-diolate (Sper/NO) (Alexis) or spermine (Sigma) was added 3 min after fertilization. Samples (50 ml) were collected at 5, 9, 24 and 48 h post fertilization (hpf) by centrifugation at 1800 rcf for 10 min in a swing out rotor at 4°C. The pellet was washed with phosphate buffered saline and then frozen in liquid nitrogen and kept at −80°C. Total RNA was extracted for each developmental stage using TRIzol (Invitrogen) according to the manufacter's instructions. Extraction with chloroform/isoamyl alcohol (24∶1) was performed following RNA precipitation by addition of glycogen and isopropyl alcohol. Contaminating DNA was degraded by treating each sample with DNase RNase-free kit (Roche) according to the manufacter's instructions. The quantity and purity of total RNA extracted was estimated by monitoring both the absorbance at 260 nm and 260/280 and 260/230 nm ratios by Nanodrop (ND-1000 UV-Vis Spectrophotometer; NanoDrop Technologies). The quality of RNA was evaluated by gel electrophoresis. Intact rRNA subunits (28S and 18S) were observed on the gel indicating minimal degradation of the RNA. For each sample 600 ng of total extracted RNA was retrotranscribed with iScript™ cDNA Synthesis kit (Biorad) following the manufacter's instructions. cDNA was diluted 1∶2 with H_2_O prior to use in Real Time qPCR experiments.

### Isolation of reference gene and Real Time qPCR

For all real time qPCR experiments the data from each cDNA sample were normalized against ubiquitin mRNA level as endogenous reference, the expression level of which remained relatively constant in all the developmental stages examined according to Nemer *et al*, 1991 [33]. Because ubiquitin sequence of *P. lividus* is not available, a 150 bp fragment was amplified using specific primers for ubiquitin of *Strongylocentrotus purpuratus* [33], [34]. The amplified fragment using Taq High Fidelity PCR System (Roche) was purified from agarose gel using QIAquick Gel extraction kit (Qiagen) and specificity of PCR product for ubiquitin was checked by DNA sequencing. The same procedure was applied in order to analyze the expression level of *NOS* gene. Because *NOS* sequence of *P. lividus* is not available, specific primers were designed for a region of 72 bp, comprising FAD-iso domain, on *NOS* sequence of *S. purpuratus*, using the program Primer3 software (http://frodo.wi.mit.edu/cgi-bin/primer3_www.cgi; [35]: NosA_For4 5′ CCACGATACTACTCCATCTC 3′; NosA_Rev5 5′ GACCACGGCGACGGTTGCATG 3′. Specificity of PCR product was checked by DNA sequencing (see above). Specific primer sets for *hsp70* (accession number X61379) [36] and *caspase-8* (accession number EU078681) [37] were designed on the basis of sequences. The following primers were used:

hsp70_forward 5′ CAGAACCACGCCCAGCTATG 3′;

hsp70_reverse 5′ GCTTGGATGCTACTATCGTTG 3′;

Cas8_Pl_F2 5′ GATACGACGAGCAGCGCAACATCTAG 3′;

Cas8_Pl_R2 5′ CTAGCATCATCCACTCTCATCCACTGCAC 3′.

A fragment of 150 bp was amplified for *hsp70* gene, a fragment of 146 bp for *caspase-8* gene.

Specificity of every amplification reaction was verified by melting curve analysis. The efficiency of each primer pair was calculated according to standard methods curves using the equation E = 10^−1/slope^. Five serial dilutions were set up to determine Ct values and reaction efficiencies for all primer pairs. Standard curves were generated for each oligonucleotide pair using the Ct values versus the logarithm of each dilution factor. PCR efficiencies were calculated for reference and target genes and were found to be 2. Diluted cDNA was used as a template in a reaction containing a final concentration of 0.3 µM for each primer and 1× FastStart SYBR Green master mix (total volume of 25 µl). PCR amplifications were performed in a Chromo 4™ Real Time Detector (Biorad) thermal cycler using the following thermal profile: 95°C for 10 min, one cycle for cDNA denaturation; 95°C for 15 sec and 60°C for 1 min, 40 cycles for amplification; 72°C for 5 min, one cycle for final elongation; one cycle for melting curve analysis (from 60°C to 95°C) to verify the presence of a single product. Each assay included a no-template control for each primer pair. To capture intra-assay variability all Real Time qPCR reactions were carried out in triplicate. Fluorescence was measured using Opticon Monitor 3.1 (Biorad). The expression of each gene was analyzed and internally normalized against ubiquitin using REST software (Relative Expression Software Tool) based on Pfaffl method [38], [39]. Relative expression ratios above two cycles were considered significant. Experiments were repeated at least twice. Statistical analysis was performed using GraphPad Prism version 4.00 for Windows (GraphPad Software, San Diego California USA).

## Results

### Involvement of NO in DD-induced apoptosis

**Figure 1:**
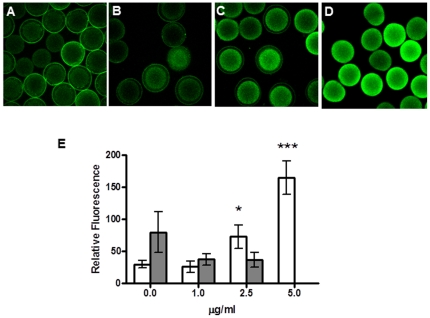
Detection of endogenous NO levels in sea urchin fertilized eggs. NO was revealed by the NO–specific indicator DAF-FM-DA. (A) Control eggs, (B,C and D) eggs treated for 10 min with 1, 2.5 and 5 µg/ml DD, respectively, and then fertilized. The images were acquired 20 min post-fertilization. (E) Relative fluorescence after 20 min post-fertilization for the same samples reported in the upper panel. White bars indicate relative fluorescence inside the eggs. Gray bars indicate fluorescence in the perivitelline space and fertilization membrane. Values are reported as mean ± S.D. *, *p*<0.05; *** *p*<0.001 with respect to the control. Statistical significance of 5 µg/ml: *** *p*<0.001 compared with 2.5 µg/ml. Control: N = 5; DD 1 µg/ml: N = 4; DD 2.5 µg/ml: N = 4; DD 5 µg/ml: N = 6.

Endogenous NO levels were monitored in *P. lividus* eggs using the NO indicator DAF-FM-DA. Control eggs produced NO soon after fertilization and after 20 min fluorescence was mainly visible in the perivitelline space and fertilization membrane (Figure 1A,E). Eggs treated with DD showed a stronger increase in NO levels within the matrix of the eggs compared to controls, and this increment was concentration-dependent (Figure 1B–E). At the highest DD concentration (see Figure 1D), the fertilization membrane adhered to the egg surface indicating that DD interfered with elevation of the membrane typical at fertilization. The NO scavenger c-PTIO led to a decrease in measured fluorescence both in the control (75%) and 3.5 µg/ml DD-treated samples (41%).

**Figure 2:**
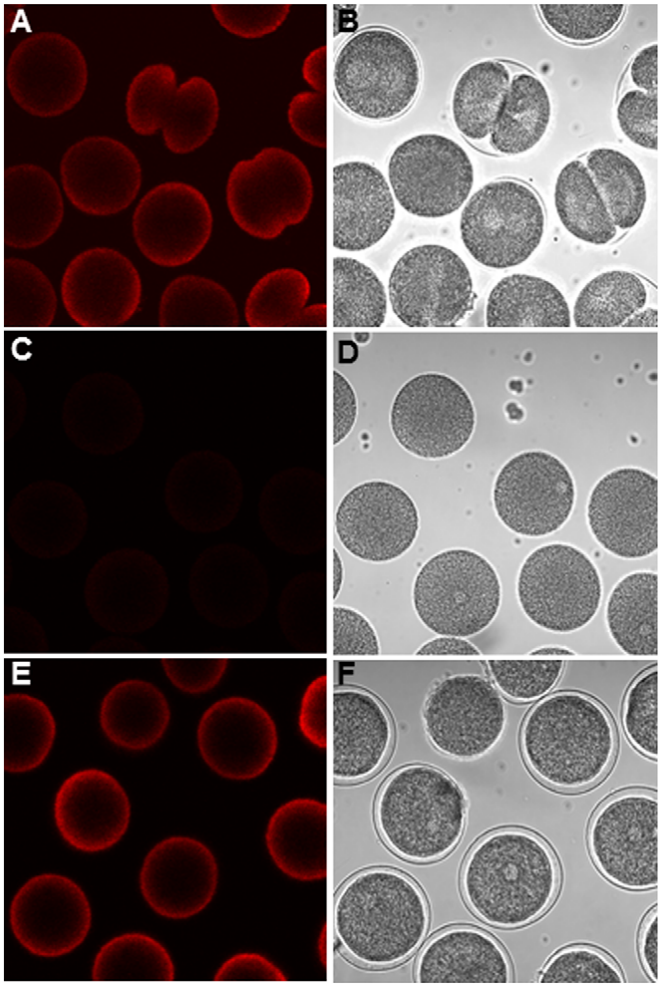
Mitocondrial functionality of developing sea urchin embryos. (A,C and E) Active mitochondria revealed by the mitochondrial-specific fluorescent dye Mitotracker after 50 min post-fertilization. (B,D and F) Corresponding bright field images. (A) Control embryos. (C) Embryos incubated with 5 µg/ml DD; (E) embryos incubated with 5 µg/ml DD in the presence of the peroxynitrite scavenger MnTBAP (10 µM).

**Figure 3:**
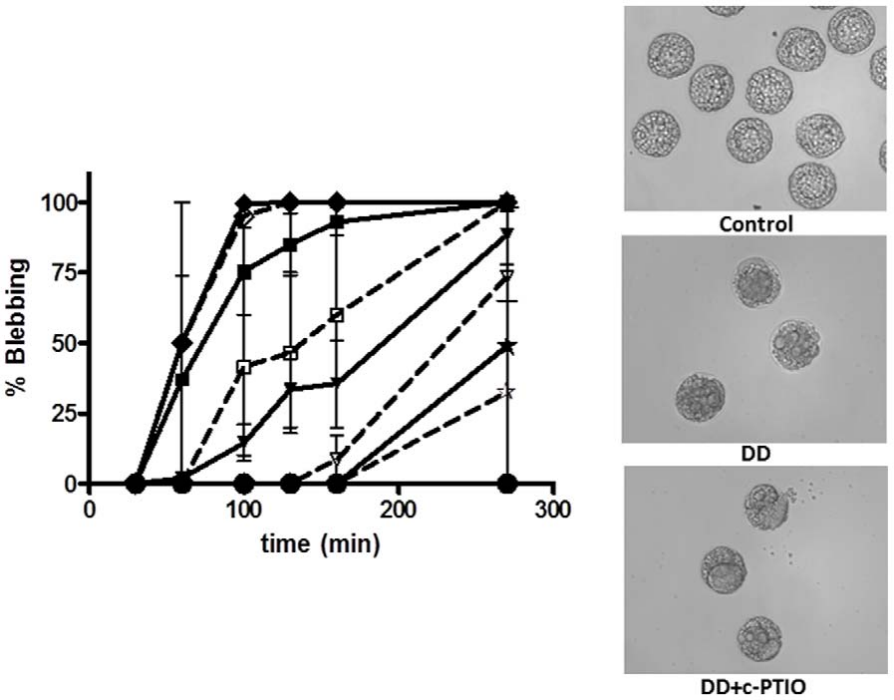
Effect of DD on the appearance of blebbing. Sea urchin embryos were treated with DD at different concentrations in the absence or presence of 800 µM c-PTIO as described in materials and method section. Left panel: embryos were monitored for blebbing appearance at 30, 60, 100, 130, 160 and 270 min after fertilization. Control (circle); DD 5 µg/ml (diamond), 2.5 µg/ml (square), 1 µg/ml (triangle), 0.5 µg/ml (star). Dashed lines with empty shapes indicate data obtained in the presence of c-PTIO. Right panel: Control and embryos treated with DD 1 µg/ml in the absence or presence of 800 µM c-PTIO observed at 270 min after fertilization. Values are reported as mean ± S.D.

High DD concentrations (>3.5 µg/ml) are known to block cell cleavage and induce apoptosis [17]. Here we investigated whether NO was involved in the induction of apoptosis by examining for the first time the effect of DD on mitochondrial functionality. After treatment with DD in the presence of Mitotracker, which specifically marks active mitochondria, fluorescence decreased dramatically with respect to the control (Figure 2A,C), thus revealing that DD impairs mitochondrial functionality. This was rescued by the peroxynitrite scavenger MnTBAP (Figure 2E) suggesting that oxidative species deriving from NO, such as peroxynitrite, were implicated in initial apoptotic pathways. To further investigate the involvement of NO in apoptosis progression, we treated unfertilized eggs with DD in the presence of the NO-scavenger c-PTIO. This treatment did not revert apoptosis since blebbing, monitored at different time intervals, occurred at all DD concentrations tested and was evident throughout the embryo (Figure 3). Similar results were obtained with the peroxynitrite scavenger MnTBAP (data not shown).

### Involvement of NO in DD-induced teratogenesis

**Figure 4:**
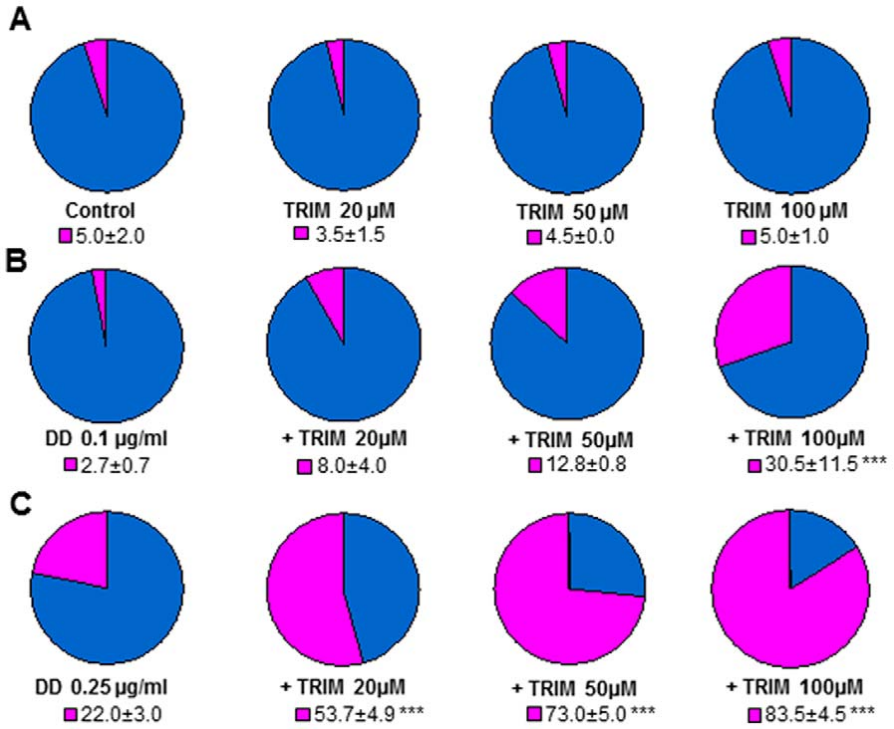
NO involvement in DD-induced teratogenesis. Embryo development was monitored after 48 hours post fertilization (hpf). (A) Control eggs and eggs treated with TRIM at 20, 50 and 100 µM. (B, C) Eggs treated with DD at 0.1 µg/ml and 0.25 µg/ml, respectively in the absence and presence of TRIM at different concentrations. *** *p*<0.001 compared to the corresponding DD concentration. Blue: normal plutei. Pink: abnormal plutei.

As shown in a previous study [11], teratogenesis in sea urchins occurs at ≥0.2 µg/ml DD with an increase in the number of abnormal plutei. These plutei showed severe malformations such as asymmetrical arms and spicules, reduced length of the arms and spicules, and a shortening of the apex from an “Eiffel Tower”-like triangular larva to a more rounded pyramidal shape as if these larvae were retarded in growth. Such larvae did not develop further and were hence considered abnormal (teratogenic) because they deviated from normal development. To investigate the possible involvement of NO in teratogenesis induced by low DD concentrations the effect of different NOS inhibitors was examined on sea urchin development. The inhibitors included the amino acid L-NA, which competes for the binding site of the substrate L-arginine, and the imidazole derivative TRIM which interferes with the binding of both L-arginine and the cofactor BH_4_. These inhibitors were first tested together with the control D-NA which is inactive on NOS. Both L-NA and D-NA affected development compared to TRIM which had no effect at the concentrations tested (Figure S1). For these reasons, TRIM was used in successive experiments. Here, we show that at two different DD concentrations (0.1, 0.25 µg/ml), the number of abnormal plutei increased with increasing TRIM concentrations (from 20 to 100 µM) suggesting a protective function for NO against teratogenesis (Figure 4).

### NO-mediated teratogenesis: gene expression

**Figure 5:**
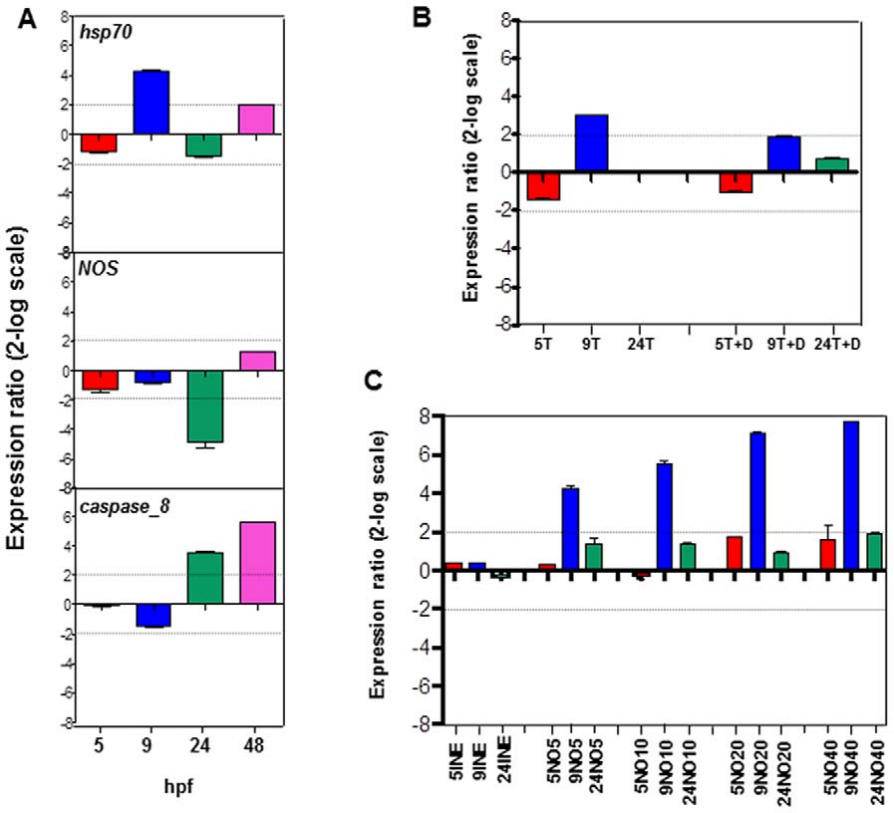
Gene expression levels using Real Time qPCR. (A) *hsp70*, *NOS* and *caspase-8* gene expression levels were followed by Real Time qPCR. Samples incubated with DD were collected at 5, 9, 24 and 48 hpf. (B) *hsp70* in samples incubated with DD in the absence and presence of TRIM. 5T, 9T, 24T: samples treated with TRIM and collected at 5, 9 and 24 hpf, respectively. 5T+D, 9T+D, 24T+D: samples treated with TRIM and DD and collected at different times. (C) *hsp70* in samples incubated with increasing concentrations of sper/NO. Spermine was used as a control. 5INE, 9INE, 24INE: samples treated with spermine at different times; 5NO5, 9NO5, 24NO5: samples treated with sper/NO 5 µM; 5NO10, 9NO10, 24NO10: samples treated with sper/NO 10 µM; 5NO20, 9NO20, 24NO20: samples treated with sper/NO 20 µM; 5NO40, 9NO40, 24NO40: samples treated with sper/NO 40 µM. Data in histograms are expressed as a fold difference from control and are reported as mean ± S.D. Fold differences greater than ±2 (see dotted horizontal guidelines at values of 2 and −2 on the histograms) were considered significant.

Expression levels of some relevant genes, such as *hsp70*, *NOS* and *caspase-8*, were followed by real time qPCR in *P. lividus* developing embryos incubated in the presence of 0.25 µg/ml DD. Samples were collected at 5, 9, 24 and 48 hpf, corresponding to the stages of early blastula, swimming blastula, prisma and pluteus, respectively. As endogenous reference we used the gene which encodes for ubiquitin, the expression of which remained constant in all examined stages. Figure 5A shows the relative expression ratio of examined genes with respect to the control. *Hsp70* showed a 4.4-fold increase at 9 hpf in the swimming blastula. At 5, 24 and 48 hpf, expression levels were comparable to the control. *NOS* gene remained at the basal level during all developmental stages except in the prisma where there was a 5.2-fold decrease in the expression of the gene compared to the control. Expression of *caspase-8* increased at 24 hpf, reaching values of 3.4 and 5.3-fold increase at 24 and 48 hpf in the prism and pluteus, respectively.

To test whether NO was involved in the increased expression of *hsp70*, eggs were pre-treated with 0.25 µg/ml DD in the presence of TRIM and then fertilized. Samples were collected after 5, 9 and 24 hpf and checked for *hsp70* expression. A 2-fold increase was recorded after 9 hpf in the swimming blastula (Figure 5B) as opposed to the 4.4-fold increase in the absence of TRIM (see Figure 5A), indicating that NO contributed to the activation of *hsp70* expression. No significant differences were observed for blastula (5 hpf) and prisma (24 hpf) stages. TRIM alone induced a 3-fold increase in *hsp* 70 expression suggesting that TRIM may generate a slight stress response in sea urchin embryos at 9 hpf.

To further demonstrate that NO is responsible for *hsp70* activation, samples of sea urchin eggs were treated with increasing concentrations of the NO donor, sper/NO. As a control we used spermine, the product deriving from sper/NO after NO release. At 9 hpf the relative expression of *hsp70* increased 4.1-, 5.4-, 7.1- and 7.6-fold at 5, 10, 20 and 40 µM sper/NO, respectively (Fig. 5C). At 5 hpf and 24 hpf there was no significant difference with respect to the control.

## Discussion

The results of this study provide evidence that NO mediates the stress response of sea urchin *P. lividus* embryos against the toxic effects of the diatom-derived aldehyde DD. At high DD concentrations (≥2.5 µg/ml), there is a dramatic burst in NO production in newly fertilized eggs compared to controls. At this stage, eggs are known to undergo a rapid increase in NO production which mobilizes intracellular calcium stores, regulates the duration of the calcium transient and fertilization envelope hardening [40], [18], [19]. In our experiments the increase in NO was mainly localized on the fertilization membrane in control conditions, whereas after treatment with DD the NO burst was mostly associated with the egg matrix. To our knowledge this is the first study to report such an effect on NO stores in the egg matrix challenged with a toxicant. With increasing DD concentrations, NO increased and at 5 µg/ml elevation of the fertilization envelope was hampered suggesting interference with exocytosis and hardening processes. This burst in NO eventually leads to initial stages of apoptosis (i.e. alterations of mitochondrial membrane potential) through the formation of the highly reactive species peroxynitrite, thus providing new insights on the mechanism of action of DD. The fact that scavengers of NO or peroxynitrite were unable to revert morphological changes typical of final stages of apoptosis, such as blebbing, suggests that other apoptotic NO-independent pathways are responsible for apoptosis progression at high DD concentrations [17].

At low DD concentrations (0.25 µg/ml), NO seems to have a protective function, acting to defend the embryo against teratogenesis by increasing *hsp70* expression levels. When NO levels are lowered by inhibiting NOS activity with TRIM, the expression of *hsp70* decreases and the proportion of abnormal plutei increases. On the other hand, increasing NO levels with the NO donor Sper/NO triggers a dose-dependent increase in *hsp70* expression. Sea urchins have been shown to activate different hsps as a general protective strategy against a variety of stress-inducing agents [41]–[43], including heat shock [44]–[46], heavy metals [45], [47], [48], and the calcium chelator EGTA [49]. Activation of hsp70 by NO has also been observed in other systems such as hepatocyte cell cultures and rat organs, in response to heat shock [50]. Here we show that sea urchins activate this hsp when challenged with DD at lower concentrations (0.25 µg/ml) at the swimming blastula stage. However, prolonged exposure to DD (24 and 48 hpf) at this concentration leads to a decrease in *hsp70* expression levels compared to controls, with a concomitant down-regulation in *NOS* levels. Hence NO is no longer able to exert a protective function and, as a consequence, at 48 hpf the expression of the initiator *caspase-8* increases.

This Janus faced (*sensu* Snyder, 1993) [51] role of NO in sea urchin recalls the “stress surveillance system” described by Vardi *et al*, 2006 [52], who observed that at high DD concentrations (≥2 µg/ml) there was a burst in NO production in the marine diatom *Phaeodactylum tricornutum* which resulted in cell death of this unicellular alga. Pretreatment of cells with sub-lethal doses of this aldehyde (0.1 µg/ml for 2 hr), however, induced resistance to subsequent lethal doses of DD. Our results showing a protective function for NO may provide a molecular explanation to this resistance due to the activation of *hsp70* expression. Other examples of this dual role of NO are reported both in plants and animals. In higher terrestrial plants NO has been shown to be either toxic or protective to abiotic stress such as heat shock, drought stress, salinity, UV-B radiation and heavy metal toxicity [53]–[59] depending on both the concentration of NO and the tissue where it is acting. Also in some human diseases such as in multiple sclerosis NO appears to have a dual function, one pro-inflammatory that triggers disease onset, and the other neuroprotective that promotes recovery from disease exacerbation events [60]. Although both could be mediated directly by NO, it is possible that the negative outcomes triggered by NO production could ensue from its conversion to the toxic metabolite peroxynitrite. This reactive molecule modifies proteins through the formation of nitrotyrosine adducts and, when present at sufficiently high levels, induces DNA damage and apoptosis [61], [62]. Overall these observations suggest that the dual role of NO could be considered as a general rule.

In marine invertebrates, studies on the role of NO in mediating stress response induced by toxic agents are scarce. NO is used as a biomarker of pollution-induced stress in marine invertebrates [63] but nothing is known as to whether this mechanism is mediated by activating hsps. Cells respond to adverse environmental stimuli, such as toxic concentrations of heavy metals, by enhancing the expression of hsps which play an important role in cellular protection [64]. The most abundant and reacting hsp to both physiological and environmental stress is hsp70 which is highly conserved during evolution and is currently used as a biomarker to monitor the biological impact of toxic chemicals on various species, including many invertebrates. For example, in sea urchins hsp70 expression has been used as a biomarker to detect exposure to pollutants due to the high correlation between contamination events and hsp70 protein levels [47], [65], [66].

Our findings that NO production increases rapidly in response to a toxic exogenous stimulus such as diatom aldehydes, and that NO induces an increase in *hsp70* expression levels, opens new perspectives on the possible role of this gas as a universal messenger to environmental stress in sea urchins. Given the importance of diatom blooms in nutrient-rich aquatic environments, our results also have important implications for understanding the cellular mechanisms underlying the responses of benthic organisms, such as sea urchins, to aldehyde exposure. Sea urchin eggs and larvae may come into contact with diatom PUAs in the field at the end of a bloom, with the mass sinking of diatoms to the sediment. Due to the patchy nature of phytoplankton at sea, it is reasonable to expect high local concentrations in the proximity of breakage of diatom cells. Ribalet [67] estimated that such concentrations were within the significant range for affecting growth and performance of surrounding organisms. Our results indicate that even low concentrations of PUAs can affect the developmental program in sea urchin embryos, with evident malformations and apoptosis induction suggesting that most of these embryos are destined to die. What remains poorly understood is if sea urchins actually feed on diatoms at the end of the bloom thereby compromising their fitness.

## Supporting Information

Figure S1 **Effect of NOS inhibitors on sea urchin development.** (A) Control. (B, C, D) L-NA at 20, 50 and 100 µM, respectively. (E, F, G) D-NA at 20, 50 and 100 µM, respectively. (H, I, J) TRIM at 20, 50 and 100 µM, respectively. The images were taken at 48 hpf.
(TIF)
