# Supplementary figures and images for: Nitric Oxide Mediates the Stress Response Induced by Diatom Aldehydes in the Sea Urchin Paracentrotus lividus
Source: PLoS One. 2011 Oct 11;6(10):e25980. doi: 10.1371/journal.pone.0025980 (PMC3191173; doi:10.1371/journal.pone.0025980)

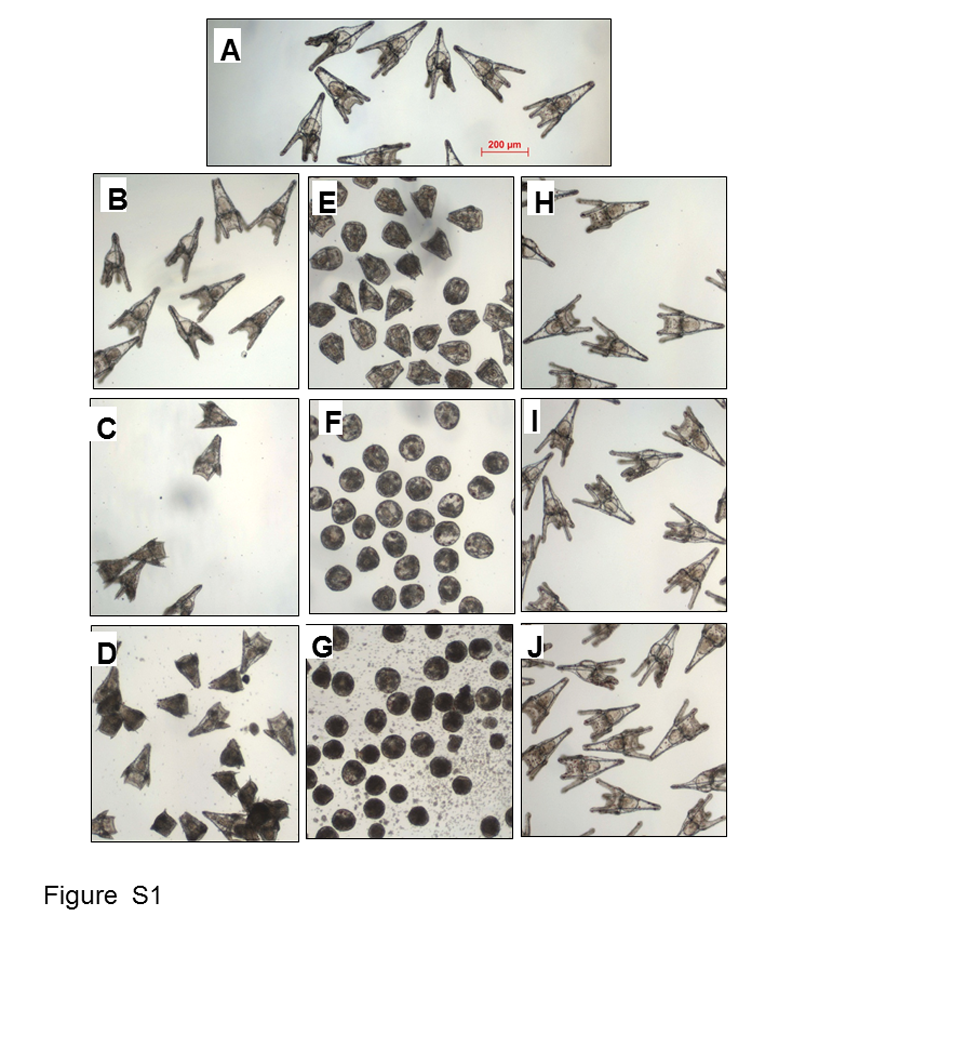

Supplement: Figure S1 — Effect of NOS inhibitors on sea urchin development. (A) Control. (B, C, D) L-NA at 20, 50 and 100 µM, respectively. (E, F, G) D-NA at 20, 50 and 100 µM, respectively. (H, I, J) TRIM at 20, 50 and 100 µM, respectively. The images were taken at 48 hpf. (TIF) [file pone.0025980.s001.tif]
